# Native T1 adds independent value for cardiovascular risk assessment beyond global longitudinal strain in an all-comers real-world clinical patient population

**DOI:** 10.1093/ehjopen/oeaf109

**Published:** 2025-08-20

**Authors:** Sören J Backhaus, Julia Treiber, Jan Sebastian Wolter, Steffen D Kriechbaum, Ulla Fischer, Andreas Schuster, Valentina O Puntmann, Eike Nagel, Samuel Sossalla, Andreas Rolf

**Affiliations:** Department of Cardiology, Campus Kerckhoff of the Justus-Liebig-University Giessen, Kerckhoff-Heart-Centre, Kerckhoff-Clinic, Benekestr. 2-8, 61231 Bad Nauheim, Germany; German Center for Cardiovascular Research (DZHK), Partner Site Rhine-Main, Kerckhoff-Clinic, Benekestr. 2-8, 61231 Bad Nauheim, Germany; Department of Cardiology and Angiology, Medical Clinic I, University Hospital Giessen, Justus-Liebig-University Giessen, Klinikstraße 33, 35392 Giessen, Germany; Department of Cardiology, Campus Kerckhoff of the Justus-Liebig-University Giessen, Kerckhoff-Heart-Centre, Kerckhoff-Clinic, Benekestr. 2-8, 61231 Bad Nauheim, Germany; German Center for Cardiovascular Research (DZHK), Partner Site Rhine-Main, Kerckhoff-Clinic, Benekestr. 2-8, 61231 Bad Nauheim, Germany; Department of Cardiology, Campus Kerckhoff of the Justus-Liebig-University Giessen, Kerckhoff-Heart-Centre, Kerckhoff-Clinic, Benekestr. 2-8, 61231 Bad Nauheim, Germany; German Center for Cardiovascular Research (DZHK), Partner Site Rhine-Main, Kerckhoff-Clinic, Benekestr. 2-8, 61231 Bad Nauheim, Germany; Department of Cardiology, Campus Kerckhoff of the Justus-Liebig-University Giessen, Kerckhoff-Heart-Centre, Kerckhoff-Clinic, Benekestr. 2-8, 61231 Bad Nauheim, Germany; German Center for Cardiovascular Research (DZHK), Partner Site Rhine-Main, Kerckhoff-Clinic, Benekestr. 2-8, 61231 Bad Nauheim, Germany; Department of Cardiology, Campus Kerckhoff of the Justus-Liebig-University Giessen, Kerckhoff-Heart-Centre, Kerckhoff-Clinic, Benekestr. 2-8, 61231 Bad Nauheim, Germany; German Center for Cardiovascular Research (DZHK), Partner Site Rhine-Main, Kerckhoff-Clinic, Benekestr. 2-8, 61231 Bad Nauheim, Germany; University Medical Center Göttingen, Department of Cardiology and Pneumology, Georg-August University, Robert-Koch-Straße 40, 37075 Göttingen, Germany; German Center for Cardiovascular Research (DZHK), Partner Site Lower Saxony, Georg-August University, Robert-Koch-Straße 40, 37075 Göttingen, Germany; FORUM Cardiology, An d. Ziegelei 1, 37124 Rosdorf, Germany; Institute for Experimental and Translational Cardiovascular Imaging, DZHK Centre for Cardiovascular Imaging, Goethe University Frankfurt, Theodor-Stern-Kai 7, 60590 Frankfurt am Main, Germany; Institute for Experimental and Translational Cardiovascular Imaging, DZHK Centre for Cardiovascular Imaging, Goethe University Frankfurt, Theodor-Stern-Kai 7, 60590 Frankfurt am Main, Germany; Department of Cardiology, Campus Kerckhoff of the Justus-Liebig-University Giessen, Kerckhoff-Heart-Centre, Kerckhoff-Clinic, Benekestr. 2-8, 61231 Bad Nauheim, Germany; German Center for Cardiovascular Research (DZHK), Partner Site Rhine-Main, Kerckhoff-Clinic, Benekestr. 2-8, 61231 Bad Nauheim, Germany; Department of Cardiology and Angiology, Medical Clinic I, University Hospital Giessen, Justus-Liebig-University Giessen, Klinikstraße 33, 35392 Giessen, Germany; Department of Cardiology, Campus Kerckhoff of the Justus-Liebig-University Giessen, Kerckhoff-Heart-Centre, Kerckhoff-Clinic, Benekestr. 2-8, 61231 Bad Nauheim, Germany; German Center for Cardiovascular Research (DZHK), Partner Site Rhine-Main, Kerckhoff-Clinic, Benekestr. 2-8, 61231 Bad Nauheim, Germany; Department of Cardiology and Angiology, Medical Clinic I, University Hospital Giessen, Justus-Liebig-University Giessen, Klinikstraße 33, 35392 Giessen, Germany

**Keywords:** Cardiovascular magnetic resonance, Deformation imaging, Tissue characterization

## Abstract

**Aims:**

Deformation imaging remains underused for cardiovascular risk assessment. As tissue characterization has now been recognized as an additional assessment tool, we sought to investigate the significance of native T1 and extracellular volume (ECV) in an unselected clinical routine population.

**Methods and results:**

The single-centre, prospective cardiovascular magnetic resonance (CMR) registry included patients referred for clinical CMR. Left ventricle global longitudinal strain (GLS) was evaluated in long-axis views. Native T1 and ECV were assessed on septal, basal, or midventricular short-axis positions. Follow-up was conducted for primary (all-cause mortality and heart failure hospitalization) and secondary (all-cause mortality, hospitalized angina, and myocardial infarction) endpoints. The final population consisted of *n* = 1633 patients who met primary (*n* = 68) and secondary (*n* = 90) endpoints during the median follow-up of 395 days. A 10-ms T1 increase was associated with a hazard ratio (HR) of 1.11 [95% confidence interval (CI) 1.07–1.15, *P* < 0.001] for the primary endpoint independent of ECV (*P* = 0.738). T1 (HR 1.07, 95% CI 1.03–1.11, *P* = 0.001) but not ECV (*P* = 0.674) was an independent predictor for the primary endpoint after correction for common risk factors including age, New York Heart Association class, biomarker NT-proBNP/glomerular filtration rate, and GLS. After dichotomization at the median of 1126 ms, T1 added incremental value for primary endpoint prediction on Kaplan–Meier plots in patients with left ventricular ejection fraction above/below (*P* = 0.019/0.017) the median of 55% and GLS above/below (*P* = 0.019/0.041) the median of −16.4%.

**Conclusion:**

Native T1 was found to be an independent risk predictor beyond GLS as well as common clinical risk factors. This may justify the use of non-contrast CMR protocols in selected patients if contrast application is contraindicated.

## Graphical Abstract

**Graphical Abstract:**
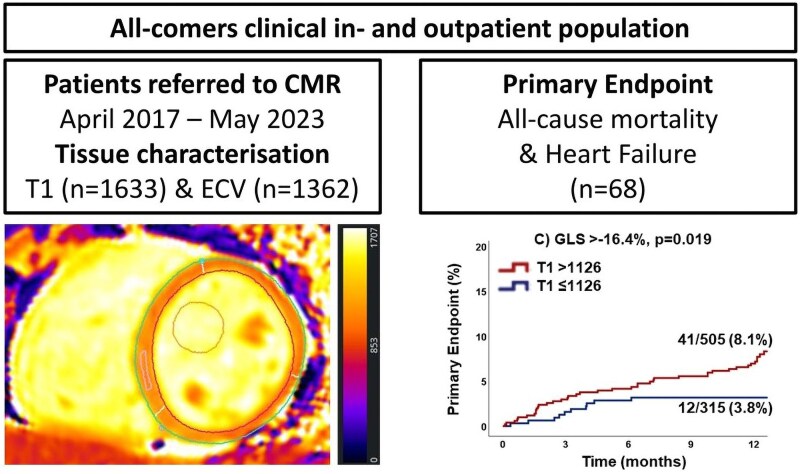

## Introduction

Cardiovascular diseases (CVDs) are the leading cause of mortality worldwide^1,2^ despite all advances made in the realms of ischaemic and non-ischaemic disease categories.^3–6^ On the one hand, optimized patient care requires early diagnosis. This is of pivotal importance in diseases where therapeutic intervention addresses primarily cardiac remodelling, e.g. in heart failure with preserved ejection fraction (HFpEF).^7^ On the other hand, individual patient management requires accurate evaluation of overall risk.^5^ Although the utility of left ventricular ejection fraction (LVEF) in risk prediction has been extensively reviewed,^4^ deformation imaging as a more appropriate approach for overall myocardial functional and risk assessment has yet to find its way into routine clinical imaging. Tedious post-processing and software-dependent reference values have been discussed as underlying reasons.^8,9^ Beyond functional quantification, tissue characterization has come to the fore. Scar tissue is recognized as a substrate for arrhythmias.^6^ Indeed, scar burden and myocardial remodelling have been introduced as characteristics for risk evaluation in cardiomyopathies^5^ or are under ongoing investigation, e.g. in dilated cardiomyopathy with LVEF >35% in the cardiovascular magnetic resonance (CMR)-ICD trial (NCT04558723).

Cardiovascular magnetic resonance is the reference standard for cardiac morphological evaluation^10^ and allows dedicated deformation assessment as well as tissue characterization. The latter is performed by the means of native T1 and T2, extracellular volume (ECV) quantification, and late gadolinium enhancement (LGE).^11^ Whilst native T1 offers an approach for tissue characterization independent of contrast application, ECV may allow for a more detailed evaluation of extracellular remodelling that covers T1 both before and after contrast application, thus incorporating the advantages of gadolinium dynamics within the tissue.^12^ A body of evidence reports the value of tissue characterization in specific CVDs;^13,14^ however, most patients are referred to CMR imaging because a specific diagnosis has yet to be obtained or the severity of CVD needs further evaluation. Consequently, we sought to evaluate the significance of tissue characterization in addition to state-of-the-art cardiac functional quantification for cardiovascular outcome in an unselected, all-comers clinical patient population.

## Methods

### Study population

**Figure 1:**
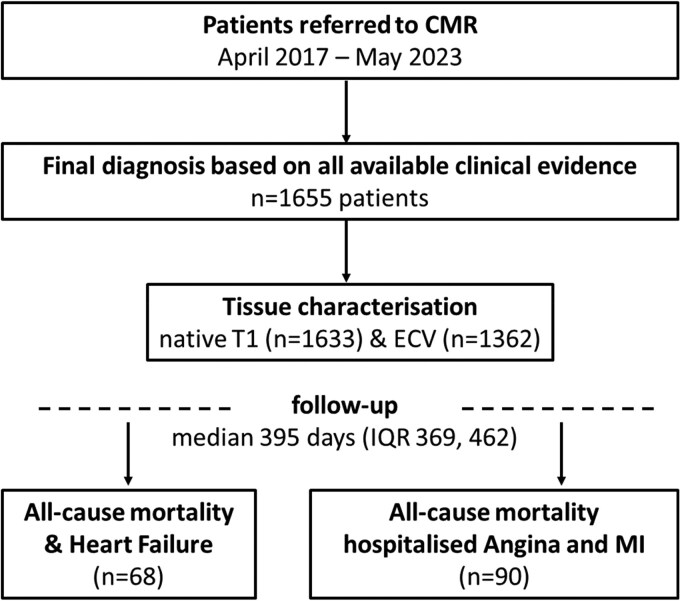
Study flow chart. CMR, cardiovascular magnetic resonance; ECV, extracellular volume; MI, myocardial infarction.

The CMR/biobank (BioCVI) registry prospectively recruited all patients who were referred to the imaging unit of the Kerckhoff Heart and Thorax Centre, Bad Nauheim, Germany, and gave informed consent between April 2017 and May 2023 (*Figure 1*). These all-comers patients were recruited independent of their clinical indication, which comprised assessment of myocardial function, ischaemia, and viability testing as well as evaluation of myocardial inflammation and various cardiomyopathies. A follow-up assessment was conducted using a standardized questionnaire 1 year after the initial scan. The registry was approved by the local ethical committee of the University of Giessen and complied with the principles of the Helsinki Declaration. All patients gave written informed consent before participation.

### Cardiovascular magnetic resonance

**Figure 2:**
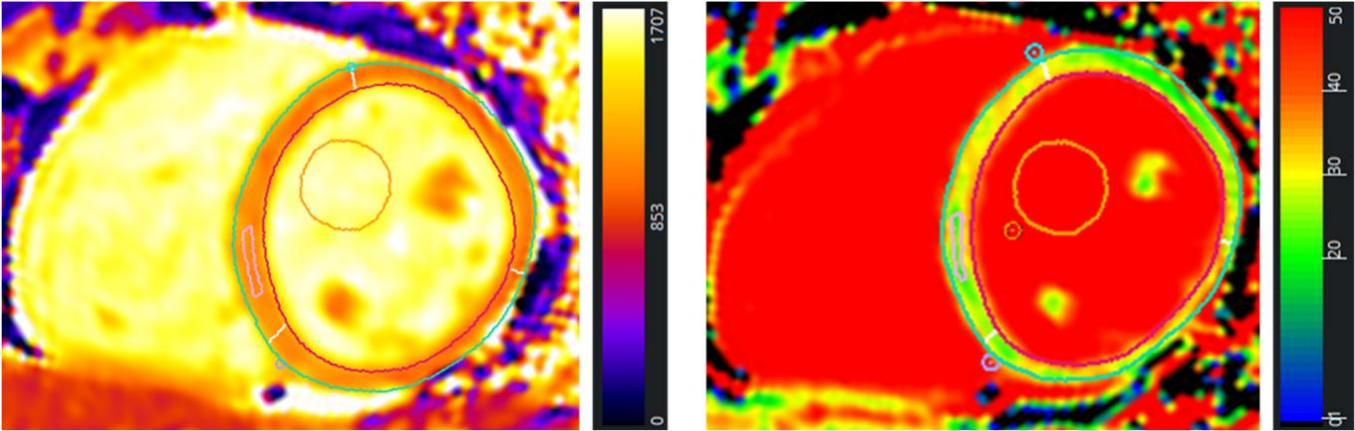
Cardiovascular magnetic resonance mapping. Exemplary assessment of septal native T1 (left) and extracellular volume (right) on a basal short-axis slice based on 5-into-3 planning.

Cardiovascular magnetic resonance imaging was conducted on a clinical 3.0 Tesla Skyra (Siemens Healthineers, Erlangen, Germany) using a 18-array coil in head-first supine position following the recommendations of the Society of Cardiovascular Magnetic Resonance.^11^ Commercially available software solutions (CVI42, Calgary, Canada) were used for post-processing. The imaging protocol included balanced steady-state free precession cine sequences in two-, three-, and four-chamber view (Ch) long-axis (LAX) orientations as well as a short-axis (SAX) stack covering both ventricles. Biventricular volumes were assessed on the SAX stack including end-diastolic/systolic and stroke volumes (EDV/ESV/SV), LVEF, and LV mass. Deformation assessment included biventricular global longitudinal strain (GLS) as well as LV global circumferential strain from LAX and SAX analyses, respectively. Tissue characterization was performed by the means of native T1 using a validated variant of a modified (3(2)3(2)5) Look-Locker imaging sequence (Goethe CVI, Frankfurt, Germany)^15^ and post-contrast T1 mapping(*Figure 2*). Subsequent calculation of ECV maps was performed if haematocrit assessment was available within 2 h of the CMR scan. Furthermore, T2 mapping and inversion-recovery-gradient echo sequences for LGE assessment of the entire SAX stack were performed 10–15 min following contrast application (Dotarem®, Guerbet, Villepinte, France; 0.15 mmol/kg bodyweight). Slice selection for mapping was based on 5-into-3 planning, and relaxation times were quantified in the centre of the basal or midventricular septum by means of a region of interest covering at least two voxels and by avoiding partial volume effects.^16^

### Clinical endpoints

The primary clinical endpoint included all-cause mortality and heart failure (HF) hospitalization in line with the American Heart Association key data elements and definitions for cardiovascular endpoints in clinical trials.^17^ The secondary endpoint included all-cause mortality and hospitalized non-fatal myocardial infarction as well as unstable angina pectoris.

### Statistics

Categorical parameters are reported as frequencies with associated percentage and were compared using the chi-squared test. Continuous parameters are reported as medians and interquartile ranges (IQR). Independent continuous parameters were compared using the Mann–Whitney *U* test. Correlations were evaluated using the Spearman’s rank correlation coefficient. Outcome analyses were based on (i) uni- and multivariate Cox proportional hazards models reported as hazard ratios (HR) with corresponding 95% confidence intervals (CIs) and (ii) Kaplan–Meier plots with associated log-rank testing. Reproducibility was assessed by the means of intraclass correlation coefficients (ICC) for absolute agreement of intra- and inter-observer measurements. All statistical evaluations considering ECV were performed in the smaller subpopulation. A two-tailed *P*-value of <0.05 was considered statistically significant. Analyses were performed with SPSS version 29.0.2.0 (IBM, Armonk, New York, USA) and MedCalc version 23.1.3 (MedCalc Software bvba, Ostend, Belgium).

## Results

### Study population

The final population consisted of *n* = 1633 patients classified as follows: Normal (*n* = 450), chronic coronary syndrome (*n* = 309), ischaemic heart disease (*n* = 275), dilated (*n* = 233), hypertrophic (*n* = 37) or other (*n* = 22) cardiomyopathies, hypertensive heart disease (*n* = 28), storage disorders (*n* = 8), inflammation (*n* = 101), acute myocardial injury (*n* = 11), right HF (*n* = 17), and others (*n* = 142). A total of *n* = 68 primary and *n* = 90 secondary endpoints were reached during the median (IQR) follow-up of 395 (369, 462) days. This was based on all-cause mortality *n* = 31, HF hospitalization *n* = 38, hospitalized non-fatal myocardial infarction *n* = 18, and unstable angina pectoris *n* = 49, whilst simultaneous occurrence of events contributed only one event in the composite endpoint. *Table 1* reports baseline characteristics for the study population. Amongst cardiovascular risk factors, hypertension and diabetes were associated with the primary (*P* = 0.032 and <0.001), whilst only hyperlipoproteinaemia was associated with the secondary (*P* < 0.001) endpoint. Coronary heart disease was more prevalent in patients with either primary or secondary endpoints (*P* < 0.001), whilst its degree in multivessel disease was similarly distributed comparing patients with no endpoint met to those who met the primary (*P* = 0.238) or secondary (*P* = 0.243) endpoint. However, previous infarction (*P* = 0.001) and percutaneous transluminal coronary angioplasty or coronary artery bypass graft (*P* < 0.001) were predominantly observed in patients who met the secondary endpoint.

**Table 1:** Baseline characteristics

Variable	All patients	No endpoint	Primary endpoint	Secondary endpoint	*P*-value
	*n* = 1633	*n* = 1514	*n* = 68	*n* = 90	
Age, y	61 (51, 71)	60 (50, 70)	69 (59,77)	70 (59, 77)	0.084/0.141
Sex, male	577/1633 (35.3)	539/1497 (35.6)	22/68 (32.4)	29/90 (32.2)	0.584/0.563
NYHA	I 130/1633 (8.0)	I 121/1514 (8.0)	I 7/68 (10.3)	I 6/90 (6.7)	<0.001/<0.001
	II 321/1633 (19.7)	II 301/1514 (19.9)	II 13/68 (19.1)	II 15/90 (16.7)	
	III 286/1633 (17.5)	III 252/1514 (16.6)	III 24/68 (35.3)	III 24/90 (26.7)	
	IV 138/1633 (8.5)	IV 115/1514 (7.6)	IV 14/68 (20.6)	IV 17/90 (18.9)	
**Cardiovascular risk factors**					
Active smoking	353/1627 (21.6)	325/1508 (21.6)	13/68 (19.1)	20/90 (22.2)	0.189/0.560
Hypertension	1007/1632 (61.7)	918/1513 (60.7)	52/68 (76.5)	65/90 (72.2)	0.032/0.065
Hyperlipoproteinaemia	779/1624 (47.7)	708/1505 (47.0)	40/68 (58.8)	60/90 (66.7)	0.132/<0.001
Diabetes	305/1628 (18.7)	272/1509 (18.0)	25/68 (36.8)	23/90 (25.6)	<0.001/0.156
Family history	565/1599 (34.6)	523/1484 (35.2)	16/67 (23.5)	35/87 (40.2)	0.153/0.397
Body mass index	26.8 (24.1, 30.4)	26.9 (24.1, 30.4)	26.6 (24.1, 31.0)	26.2 (24.2, 29.3)	0.985/0.281
**History of coronary heart disease**					
Coronary heart disease	604/1625 (37.0)	534/1510 (35.4)	41/67 (60.3)	59/88 (67.0)	<0.001/<0.001
Sclerosis	86	77	8	7	
1-Vessel	132	121	7	10	
2-Vessel	127	117	3	8	
3-Vessel	198	168	15	25	
Unspecified	61	51	7	9	
Previous infarction	318/1625 (19.5)	281/1508 (18.6)	20/68 (29.4)	28/88 (30.7)	0.075/0.001
Previous PTCA	385/1632 (23.6)	338/1513 (22.3)	25/68 (36.8)	40/90 (44.4)	0.021/<0.001
Previous CABG	99/1633 (6.1)	84/1514 (5.5)	7/68 (10.3)	13/90 (14.4)	0.100/<0.001
Sudden cardiac death	77/1620 (4.7)	69/1503 (4.6)	5/67 (7.4)	6/89 (6.7)	0.241/0.034
Atrial fibrillation	359/1626 (22.0)	315/1508 (20.9)	26/68 (38.2)	35/89 (39.3)	0.003/<0.001
Systolic blood pressure	125 (116, 138)	125 (116, 138)	120 (110, 136)	125 (114, 135)	0.381/0.986
Diastolic blood pressure	80 (70, 83)	80 (70, 84)	70 (63, 80)	73 (61, 80)	0.014/0.036
**Laboratory testing**					
NT-proBNP	247 (82, 896)	234 (80, 798)	1736 (588, 4506)	593 (108, 1652)	<0.001/0.004
eGFR	92 (72, 109)	92 (73, 109)	67 (46, 95)	83 (56, 101)	<0.001/0.002
**Medication**					
Beta blocker	837/1630 (51.3)	761/1511 (50.4)	45/68 (66.2)	56/90 (62.2)	0.036/0.101
ACE inhibitor	459/1628 (28.1)	419/1509 (27.8)	23/68 (33.8)	32/90 (35.6)	0.494/0.305
AT1 antagonist	378/1629 (23.1)	349/1510 (23.1)	13/68 (19.1)	22/90 (24.4)	0.681/0.836
Sacubitril/valsartan	60/1226 (3.7)	53/1133 (4.7)	5/49 (10.2)	4/70 (5.7)	<0.001/0.738
Aldosterone antagonist	228/1630 (14.0)	196/1511 (13.0)	23/68 (33.8)	19/90 (21.1)	<0.001/0.135
Ca channel blocker	254/1630 (15.6)	233/1511 (15.4)	14/68 (20.6)	14/90 (15.6)	0.483/0.913
Loop diuretic	355/1630 (21.7)	300/1511 (19.9)	41/68 (60.3)	37/90 (41.1)	<0.001/<0.001
Statin	626/1630 (38.3)	566/1511 (37.5)	33/68 (48.5)	52/90 (57.8)	0.171/<0.001
ASS	551/1629 (33.7)	504/1511 (33.4)	25/67 (36.8)	39/89 (43.8)	0.099/0.040
Malignancy <5 years	98/1627 (6.0)	87/1510 (5.8)	9/68 (13.2)	8/88 (9.1)	0.040/<0.001

Data are presented as *n*/*N* (%) or median (IQR). *P*-values were calculated for the comparison between patients with and without MACE. NT-proBNP in pg/mL.

NYHA, New York Heart Association; PTCA, percutaneous transluminal coronary angioplasty; CABG, coronary artery bypass graft; eGFR, estimated glomerular filtration rate (mL/min/1.73 m²); ACE, angiotensin-converting enzyme; AT1, Angiotensin II receptor type 1; Ca, calcium; NT-proBNP, *N*-terminal fragment of pro-brain natriuretic peptide; ASS, acetylsalicylic acid.

### Cardiac functional evaluation

*Table 2* reports echocardiographic and CMR-derived functional characteristics. All LV volumetric and deformation-based parameters were altered in patients with a primary event (*P* = 0.014−<0.001). In contrast, only LV functional parameters were impaired in patients with a secondary event (SV *P* = 0.028, EF *P* = 0.027, GLS *P* = 0.003). RV systolic function as characterized by SV (*P* = 0.022), EF (*P* < 0.001), and GLS (*P* < 0.001) was impaired in patients who met the primary but not the secondary endpoint.

**Table 2:** Functional assessments

Variable	All patients	No endpoint	Primary endpoint	Secondary endpoint	*P*-value
	*n* = 1633	*n* = 1514	*n* = 68	*n* = 90	
**Echocardiography**					
Aortic valve stenosis	0 811	0 745	0 39	0 46	0.001/0.005
	I 25	I 23	I 2	I 1	
	II 18	II 13	II 4	II 4	
	III 14	III 14	III 0	III 0	
Aortic valve regurgitation	0 694	0 644	0 30	0 33	0.055/0.202
	I 156	I 138	I 11	I 15	
	II 17	II 14	II 3	II 2	
	III 2	III 2	III 0	III 0	
Mitral valve stenosis	0 846	0 779	0 39	0 47	0.004/0.039
	I 10	I 9	I 1	I 1	
	II 5	II 3	II 2	II 2	
	III 3	III 3	III 0	III 0	
Mitral valve regurgitation	0 478	0 442	0 20	0 27	<0.001/0.502
	I 354	I 326	I 16	I 19	
	II 35	II 27	II 8	II 4	
	III 9	III 7	III 2	III 1	
Tricuspid valve regurgitation	0 511	0 476	0 23	0 23	<0.001/0.019
	I 318	I 291	I 13	I 23	
	II 24	II 20	II 4	II 2	
	III 9	III 4	III 5	III 2	
**Cardiovascular magnetic resonance imaging**					
Left ventricle					
LV Mass	51 (41, 64)	51 (41, 63)	60 (46, 73)	53 (42, 64)	0.014/0.543
LV EDV	82 (69, 102)	81 (68, 101)	97 (75, 124)	84 (70, 107)	<0.001/0.700
LV ESV	36 (27, 52)	35 (26, 50)	49 (33, 93)	39 (28, 55)	<0.001/0.175
LV SV	43 (35, 50)	43 (36, 50)	39 (28, 47)	40 (31, 49)	<0.001/0.028
LV EF	55 (44, 63)	56 (45, 63)	41 (24, 55)	52 (37, 61)	<0.001/0.027
FT LV GLS	−16.4 (−12.3, −19.3)	−16.6 (−12.6, −19.4)	−11.8 (−7.0, −15.5)	−14.3 (−11.5, −18.1)	<0.001/0.003
FT LV GCS	−17.5 (−13.1, −20.9)	−17.7 (−13.4, −21.0)	−12.5 (−7.8, −17.0)	−16.7 (−11.9, −20.1)	<0.001/0.084
Right ventricle					
RV EDV	78 (65, 93)	78 (65, 93)	81 (65, 106)	78 (66, 95)	0.164/0.829
RV ESV	38 (29, 50)	38 (29, 50)	45 (35, 60)	40 (30, 52)	0.002/0.244
RV SV	39 (32, 46)	39 (32, 46)	34 (25, 45)	36 (31, 45)	0.022/0.147
RV EF	50 (44, 57)	51 (44, 57)	45 (38, 53)	49 (43, 56)	<0.001/0.098
FT RV GLS*	−23.8 (−19.0, −27.5)	−24.0 (−19.4, −27.6)	−17.6 (−13.4, −23.6)	−22.4 (−15.1, −27.3)	<0.001/0.106
Tissue characterization					
Native T1	1126 (1090, 1169)	1125 (1089, 1167)	1168 (1119, 1238)	1133 (1096, 1188)	<0.001/0.096
ECV	25.5 (23.2, 28.3)	25.5 (23.2, 28.2)	28.0 (25.0, 33.4)	26.5 (23.8, 29.1)	<0.001/0.175

Data are presented as *n/N* (%) or median (IQR). *n = 1060. *P*-values were calculated for primary/secondary endpoint compared to no endpoint met. Volumes are given in mL/m² body surface area, EF, strain and ECV in % and T1 in ms.

EDV/ESV/SV, end-diastolic/systolic/stroke volume; EF, ejection fraction; FT, feature tracking; LV/RV, left/right ventricle; GLS/GCS, global longitudinal/circumferential strain; ECV, extracellular volume.

### Tissue characterization

Native T1 was significantly increased in patients with a primary (1168 vs. 1125, *P* < 0.001) but not secondary (1133 vs. 1125, *P* = 0.096) endpoint compared with patients without an event. Extracellular volume was available in 1362/1633 of patients only due to the absence of haematocrit assessment within 2 h of the scan. An increase in ECV was observed in patients who had a primary (28.0 vs. 25.5%, *P* < 0.001) but not in those with a secondary (26.5 vs. 25.5%, *P* = 0.175) endpoint event. There were a moderate correlation between native T1 and ECV (*r* = 0.51, *P* < 0.001) and a mild correlation of native T1 with GLS (*r* = 0.31, *P* < 0.001) and LVEF (r = −0.29, *P* < 0.001). Correlation of ECV with GLS (*r* = 0.18, *P* < 0.001) and LVEF (*r* = −0.16, *P* < 0.001) was weak but significant. Reproducibility of both T1 (ICC intra- and inter-observer 0.95) and ECV (ICC intra-observer 0.94 and inter-observer 0.91) assessments was excellent.

### Outcome

**Figure 3:**
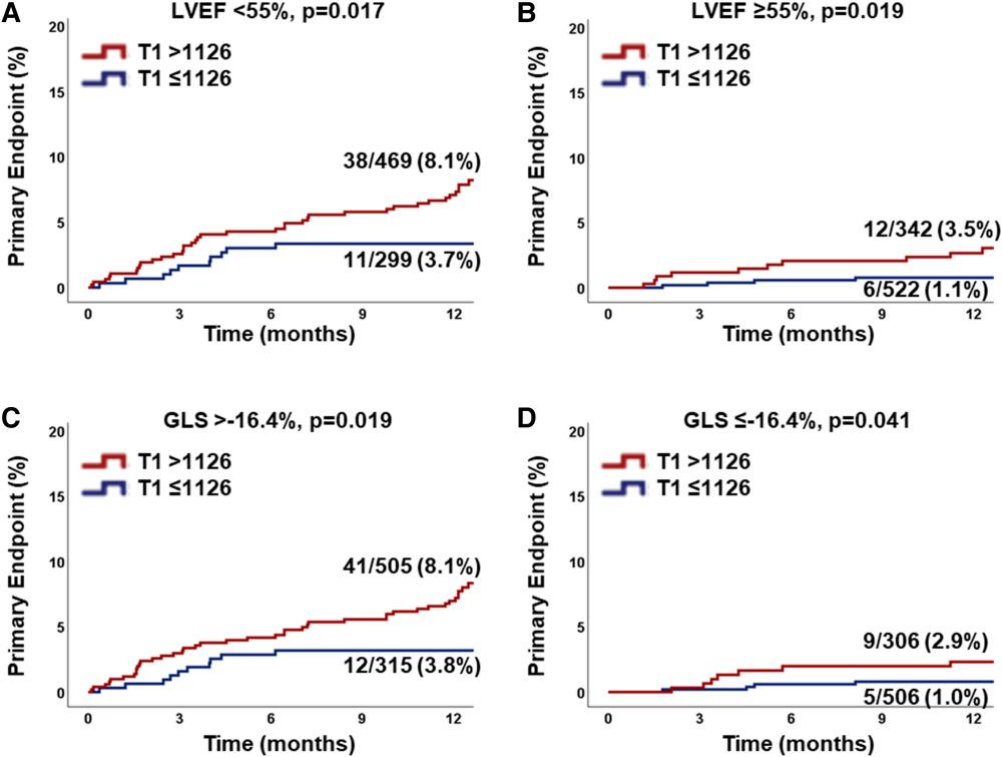
Kaplan–Meier plot—primary endpoint. (*A*) The graph shows the percentage of patients with left ventricular ejection fraction below the median of 55% who met the primary endpoint dichotomized according to the median of native T1. (*B*) The graph shows the percentage of patients with left ventricular ejection fraction above the median of 55% who met the primary endpoint dichotomized according to the median of native T1. (*C*) The graph shows the percentage of patients with global longitudinal strain above the median of −16.4% who met the primary endpoint dichotomized according to the median of native T1. (*D*) The graph shows the percentage of patients with global longitudinal strain below the median of −16.4% who met the primary endpoint dichotomized according to the median of native T1.

*Table 3* reports results from uni- and multivariate Cox regression models for the primary and secondary endpoints as well as supplementary material online, *Table S1* for all-cause mortality and HF hospitalization separately. An increase of 10 ms in native T1 or 1% in ECV was associated with an elevated HR for the primary (T1 1.10, 95% CI 1.06–1.13, *P* < 0.001 and ECV 1.02, 95% CI 1.00–1.04, *P* = 0.015) and for the secondary endpoint (T1 1.04, 95% CI 1.01–1.07, *P* = 0.018 and ECV 1.02, 95% CI 1.00–1.03, *P* = 0.046). T1 (HR 1.11, 95% CI 1.07–1.15, *P* < 0.001) but not ECV (HR 1.00, 95% CI 0.98–1.03, *P* = 0.738) was an independent predictor for the primary endpoint, whilst neither predicted the secondary endpoint independently (T1 HR 1.03, 95% CI 0.99–1.07, *P* = 0.119, ECV HR 1.01, 95% CI 0.99–1.03, *P* = 0.171). T1 (HR 1.07, 95% CI 1.03–1.11, *P* = 0.001) but not ECV (*P* = 0.142) was an independent predictor for the primary endpoint after correction for commonly considered risk factors including age, NYHA class, biomarker NT-proBNP/glomerular filtration rate, and cardiac function using GLS and tissue characterization T1 and ECV. After dichotomization at the median of 1126 ms, T1 added incremental value for primary endpoint prediction on Kaplan–Meier plots and associated log-rank testing in patients with LVEF above (*P* = 0.019) and below (*P* = 0.017) the median of 55% as well as GLS above (*P* = 0.019) and below (*P* = 0.041) the median of −16.4% (*Figure 3*).

**Table 3:** Risk assessment

Variable	Univariate primary EP	Multivariate primary EP	Univariate secondary EP	Multivariate secondary EP
**Clinical factors**				
Age	1.04 (1.02–1.06) *P* < 0.001	1.00 (0.98–1.02) *P* = 0.656	1.04 (1.02–1.06) *P* < 0.001	1.02 (1.00–1.04) *P* = 0.041
NYHA	1.63 (1.37–1.93) *P* < 0.001	1.39 (1.12–1.73) *P* = 0.003	1.36 (1.17–1.58) *P* < 0.001	1.29 (1.08–1.56) *P* = 0.006
**Laboratory testing**				
eGFR	0.98 (0.97–0.99) *P* < 0.001	0.99 (0.98–1.00) *P* = 0.089	0.99 (0.98–1.00) *P* = 0.002	1.00 (0.99–1.01) *P* = 0.619
NT-proBNP	1.00 (1.00–1.00) *P* < 0.001	1.00 (1.00–1.00) *P* = 0.431	1.00 (1.00–1.00) *P* = 0.002	1.00 (1.00–1.00) *P* = 0.243
**Echocardiography**				
Aortic valve stenosis	1.00 (0.99–1.02) *P* = 0.550		1.00 (0.99–1.01) *P* = 0.800	
Mitral valve regurgitation	1.00 (0.99–1.02) *P* = 0.459		1.00 (0.99–1.01) *P* = 0.633	
**Cardiovascular magnetic resonance**				
LVEF	0.96 (0.95–0.97) *P* < 0.001	*	0.99 (0.97–1.00) *P* = 0.068	
GLS	1.15 (1.10–1.21) *P* < 0.001	1.08 (1.02–1.15) *P* = 0.009	1.06 (1.02–1.11) *P* = 0.005	0.99 (0.94–1.05) *P* = 0.815
Native T1 (per 10 ms)	1.10 (1.06–1.13) *P* < 0.001	1.07 (1.03–1.12) *P* = 0.001	1.04 (1.01–1.07) *P* = 0.018	1.01 (0.96–1.05) *P* = 0.763
ECV (per %)	1.02 (1.00–1.04) *P* = 0.015	1.01 (0.98–1.03) *P* = 0.674	1.02 (1.00–1.03) *P* = 0.046	1.01 (0.99–1.03) *P* = 0.437

The data are presented as hazard ratios with associated 95% confidence intervals in parentheses. Variables with univariate significance (*P* < 0.05) were included in multivariable Cox regression models. *Due to colinearity of functional parameters, only GLS was considered as a CMR-derived functional parameter in multivariable models.

NYHA: New York Heart Association; eGFR, estimated glomerular filtration rate; LVEF, left ventricular ejection fraction; GLS, global longitudinal strain; GLS, global longitudinal strain; NT-proBNP, *N*-terminal fragment of pro-brain natriuretic peptide.

## Discussion

The results from this prospectively recruited registry demonstrate the independent prognostic value of CMR-derived tissue characterization in an unselected, real-world, cardiological in- and outpatient setting. Firstly, both native T1 and ECV were predictors for cardiovascular outcome. However, native T1 but not ECV emerged as an independent predictor for the primary endpoint. Secondly, native T1 remained an independent predictor for the primary endpoint regardless of clinical, laboratory, functional, and tissue characterization markers. Lastly, T1 provided incremental value to LVEF or GLS dichotomized at their respective medians. These results underline the importance of non-invasive CMR-derived tissue characterization in addition to functional quantifications.

### Tissue characterization in cardiovascular disease

Cardiovascular disease should not be regarded as a clearly circumscribed disease but rather as a complex interaction of risk factors and genetic influences that lead to inflammatory processes and multi-organ damage.^18,19^ The present study population reports a cross section of CVD in 1633 consecutively recruited cardiological in- and outpatients. This inhomogeneous group thus comprised patients classified as normal as well as those with ischaemic and non-ischaemic CVD, including inflammation or rare storage diseases.

Subclinical inflammatory processes, myocardial remodelling, and infiltration yet to show their impact on myocardial functional analyses can be detected by CMR-derived tissue characterization.^20^ Indeed, tissue characterization has demonstrated its prognostic impact^21^ in a variety of cardiovascular disorders, including coronary artery disease,^22^ non-ischaemic cardiomyopathy,^23^ and specific infiltrative disease such as amyloidosis.^24^ T1 and ECV allow detection of diffuse fibrosis as a sign of myocardial remodelling and infiltrative disease.^20^ As diffuse fibrosis is considered reversible, e.g. following catheter ablation in atrial fibrillation,^25^ its presence may be of interest for decision-making regarding therapeutic interventions. In the presence of preserved LVEF, T1 and ECV mapping can detect marked alterations in HFpEF patients^26^ and patients with systemic inflammation such as rheumatoid arthritis.^27^ Accordingly, in our cohort both T1 and ECV were predictors for the primary (all-cause mortality and HF hospitalization) and secondary (all-cause mortality and hospitalized non-fatal myocardial infarction and unstable angina pectoris) endpoints across the entire spectrum of CVD.

### Considerations of T1 vs. extracellular volume

Native T1 is affected both by the acquisition sequence and even more so by field strength.^28^ T1 showed substantial variations in a multicentre study on aortic stenosis patients across 10 centres and failed to demonstrate an association with outcome despite correction for local reference values.^12^ In a meta-analysis of 69 studies, ECV emerged as being less influenced by methodology,^28^ which seems plausible as pre- and post-contrast maps are used for calculation of ECV and thus cancel out the impact of differing T1 times between different scanners and scan environments. Indeed, ECV is associated with outcome in patients with aortic valve stenosis following aortic valve replacement.^12^ However, in our study T1 but not ECV emerged as an independent predictor for the primary endpoint. Furthermore, T1 but not ECV emerged as an independent predictor of the primary endpoint considering clinical (age and symptom severity/NYHA class), laboratory (eGFR and NT-proBNP), functional (GLS), and tissue characterization (native T1 and ECV) parameters on multivariate analyses. An underlying reason may be inherent to ECV calculation, which is based on a composite of pre- and post-contrast maps as well as bloodpool signal and haematocrit assessment. All five different assessments introduce an intrinsic variability of underlying measurements^29^ that in turn can result in increased variability in calculated ECV compared with singular native T1 assessment. Across this unselected clinical patient population, this could have resulted in a loss of significance in the light of the range of different clinical risk factors considered. Notwithstanding, inter-study reproducibility is reported to be high for both ECV^30^ and native T1.^31^ In line, in the present population, intra- and inter-observer reproducibility is high for both native T1 and ECV in the present population.

### Tissue characterization in the context of left ventricle function

Left ventricular ejection fraction is commonly used for HF classification and has persisted as the leading parameter for cardiovascular risk assessment over the past decades despite the introduction of HFpEF.^4,32^ Different subgroups with preserved LVEF have since been described, and the range of 50–60% has been associated with impaired contractility and increased ventricular fibrotic remodelling, whilst the range >60% showed less fibrotic remodelling.^33^ This suggests additional clinically prognostic relevant information may be derived from tissue characterization.

Deformation assessment—notably GLS—has demonstrated incremental prognostic value across the entirety of the HF spectrum.^34^ Tedious post-processing and vendor-specific reference ranges have slowed clinical implementation of GLS.^8,9,35^ Interestingly, the median GLS of −16.4% in the present study population fits narrowly within the area of lower limits of normal based on the published literature on speckle-tracking echocardiography as well as CMR-FT-derived strain.^36–38^ On the one hand, this may introduce −16% as a potential threshold for clinical decision-making, which would need further prospective validation. On the other hand, native T1 provided incremental prognostic value in patients below and above the medians of LVEF (55%) and GLS (−16.4%). As the present median of GLS falls within the spectrum of lower limits of normal, this finding may underline the clinical usefulness of tissue characterization for overall risk evaluation even before final diagnosis of specific CVD in addition to functional quantification. The combination of tissue characterization and functional characterization by deformation imaging would allow detailed risk assessment. Furthermore, clinical follow-up may be monitored in the absence of contrast protocols, which may be useful in repetitive imaging. The healing of myocarditis can be monitored without contrast application using native T1 and T2.^39^ As a consequence, non-contrast protocols may emerge as being suitable in selected patients.

### Limitations

The conclusions made in the present study are based on an observational registry with its associated general limitations. Nevertheless, both in- and outpatients were prospectively recruited to the registry. On the one hand, the prospective inclusion of all referred patients with informed consent allowed an analysis across a broad range of CVDs; however, on the other hand, inevitably this also leads to low generalizability of the findings to specific CVDs. Exclusion of inpatients unable to undergo CMR imaging in a supine position for 30 min may pose an inherent selection bias. The low number of endpoints reached in this real-world in- and outpatient setting may have impacted statistical power; however, multivariate models were used accordingly to avoid overfitting. Finally, the difference in patient numbers for T1 and ECV may impact their prognostic power differently and may inherently represent a selection bias; however, these cases were excluded due to unavailability of haematocrit assessment within 2 h of CMR but not specific patient characteristics. Nevertheless, multivariate analyses considered only patients with both T1 and ECV results present. In this unselected real-world clinical population, a follow-up of 1 year may be insufficient to capture all events associated with alterations in cardiac function and tissue composition.

## Conclusions

Native T1 emerges as an independent risk predictor compared to clinical, laboratory, and functional parameters in an all-comers cardiological in- and outpatient setting. Furthermore, T1 adds independent prognostic value to functional LVEF and GLS quantification. This finding may justify non-contrast CMR protocols for risk assessment in selected patients when contrast application is contraindicated.

## Lead author biography


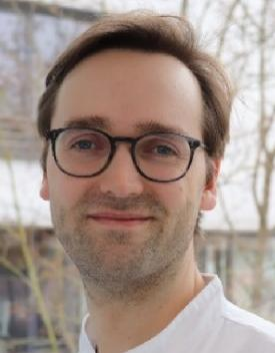


Dr Sören J. Backhaus is a consultant cardiologist at Kerckhoff Heart Centre Bad Nauheim and senior lecturer at Justus-Liebig-University Giessen. He completed his cardiology training at University Medical Center Göttingen, where he became consultant cardiologist and senior lecturer in Internal Medicine and Cardiology prior to the acceptance of his role in Bad Nauheim. He specializes in multimodality cardiovascular imaging with particular interest in cardiovascular magnetic resonance imaging in heart failure.

## Supplementary Material

oeaf109_Supplementary_Data

## Data Availability

The authors confirm that all relevant data are provided in the manuscript and all data underlying the findings are fully available without restriction and can be accessed at the Kerckhoff Heart Research Institute (KHFI) by researchers who meet the criteria for access to confidential data.
