# Supplementary material for: Native T1 adds independent value for cardiovascular risk assessment beyond global longitudinal strain in an all-comers real-world clinical patient population
Source: Eur Heart J Open. 2025 Aug 20;5(5):oeaf109. doi: 10.1093/ehjopen/oeaf109 (PMC12405752; doi:10.1093/ehjopen/oeaf109)
Supplement: oeaf109_Supplementary_Data [file oeaf109_supplementary_data.docx]

| **Table S1 Risk assessment** | | | | |
| --- | --- | --- | --- | --- |
| Variable | Univariate All-cause mortality  (n=31) | Multivariate All-cause mortality | Univariate HF-Hospitalisation  (n=37) | Multivariate HF-Hospitalisation |
| **Clinical factors** | | | | |
| Age | 1.06 (1.03-1.10) p<0.001 | 1.02 (0.99-1.06) p=0.176 | 1.02 (1.00-1.04) p=0.099 |  |
| NYHA | 1.62 (1.26-2.09) p<0.001 | 1.51 (1.11-2.03) p=0.008 | 1.61 (1.28-2.02) p<0.001 | 1.19 (0.90-1.58) p=0.234 |
| **Laboratory testing** | | | | |
| eGFR | 0.97 (0.96-0.99) p<0.001 | 0.98 (0.97-1.00)  p=0.031 | 0.99 (0.97-1.00) p=0.010 | 1.00 (0.98-1.01) p=0.584 |
| NT-proBNP | 1.00 (1.00-1.00) p=0.034 | 1.00 (1.00-1.00) p=0.612 | 1.00 (1.00-1.00) p<0.001 | 1.00 (1.00-1.00) p=0.633 |
| **Echocardiography** | | | | |
| Aortic valve stenosis | 1.01 (1.00-1.03) p=0.093 |  | 0.99 (0.97-1.01) p=0.422 |  |
| Mitral valve regurgitation | 1.01 (1.00-1.03) p=0.068 |  | 0.99 (0.97-1.02) p=0.455 |  |
| **Cardiovascular magnetic resonance** | | | | |
| LVEF | 0.98 (0.96-1.00) p=0.027 | * | 0.95 (0.93-0.97) p<0.001 | * |
| GLS | 1.08 (1.02-1.16) p=0.016 | 1.00 (0.92-1.09) p=0.927 | 1.22 (1.14-1.30) p<0.001 | 1.19 (1.09-1.29) p<0.001 |
| Native T1 (per 10ms) | 1.08 (1.03-1.13) p=0.003 | 1.05 (0.99-1.11) p=0.087 | 1.11 (1.07-1.15) p<0.001 | 1.07 (1.02-1.13) p=0.008 |
| ECV (per %) | 1.02 (0.99-1.04)  p=0.196 |  | 1.02 (1.00-1.04)  p=0.068 |  |
| The data is presented as hazard ratios with associated 95% confidence intervals in parentheses. Variables with univariate significance (p<0.05) were included in multivariable Cox regression models. Due to co-linearity of functional parameters, only GLS was considered as a CMR-derived functional parameter in multivariable models. NYHA: New York Heart Association, eGFR: estimated glomerular filtration rate, LVEF: left ventricular ejection fraction, GLS = global longitudinal strain, GLS = global longitudinal strain, NT-proBNP = N-terminal fragment of pro-brain natriuretic peptide. | | | | |
